# Identification and Expression Analysis of Minichromosome Maintenance Proteins in the Silkworm, *Bombyx mori*

**DOI:** 10.1673/031.010.14108

**Published:** 2010-09-10

**Authors:** Kohji Yamamoto, Yoichi Aso, Hiroshi Fujii

**Affiliations:** Laboratories of Insect Genetic Resources, Faculty of Agriculture, Kyushu University, 6-10-1 Hakozaki, Higashi-ku, Fukuoka 812-8581, Japan

**Keywords:** DNA replication, helicase

## Abstract

The minichromosome maintenance protein (MCM) family is involved in the regulatory role of DNA replication in eukaryotic organisms. A cDNA encoding of an MCM of the silkworm, *Bombyx mori* L. (Lepidoptera: Bombycidae), was cloned by reverse transcriptase-polymerase chain reaction (RT-PCR) and sequenced. The resultant amino acid sequence and phylogenetic analysis revealed high identity to MCM, and specifically to MCM7, of vertebrates and invertebrates. An RT-PCR showed that the bmMCM7 transcript was present in the ovaries, testes, silk glands, and fat bodies of larval silkworms. Expression plasmids were transformed into competent *Escherichia coli* and overexpressed. This is the first report on the identification of MCM helicase of the silkworm, *B. mori*.

## Introduction

The silkworm, *Bombyx mori* L. (Lepidoptera: Bombycidae), is an agriculturally important insect for silk production. More than 450 silkworm mutants have been reported, according to egg-shape, skin color at the larval stage, and cocoon-shape (http://kaiko.kyushu-u.ac.jp/index.html). Thus, the genetic resources for *B. mori* make it possible to be used as a model animal for lepidopteran insects.

Recently, an artificial fertilization technique has been applied in silkworms for the frozen storage of silkworm genetic resources without rearing (Takemura et al. 2006). Knowledge of the cell cycle, including DNA replication initiation of *B. mori,* has not been reported so far. As an initial step toward understanding DNA replication initiation of *B. mori,* an attempt was made in this study to identify factors involved in the DNA replication in silkworms. The minichromosome maintenance (MCM) gene was originally identified in *Saccharomyces cerevisiae* and was shown to participate in the initiation of DNA replication in autonomous replication sequences (ARS) (Tye 1994). MCMs were found to be responsible for the initiation of DNA replication in ARS. A number of MCM homologues have been identified. The MCM family includes six MCM proteins: MCM2, MCM3, MCM4, MCM5, MCM6, and MCM7 (Mewes et al. 1997). Six MCMs have been found in humans, mice, and frogs, suggesting that all eukaryotes contain six MCMs. Using sequence similarity, it has been reported that in *Drosophila* there are three family members, including DmMCM2, DmMCM4, and DmMCM5, and two short PCR sequences in the silkworm, *B. mori* (Feger et al. 1995; Treisman et al. 1995; Su et al. 1997). In the present study, the homologue of the cDNA sequence of DmMCM7 was identified. Furthermore, the expression pattern of the RNA encoding the putative MCM was determined by RT-PCR.

## Materials and Methods

### Insects and tissue dissection

Fifth-instar larvae of the silkworm, *B. mori*, were reared on mulberry leaves at the Institute of Genetic Resources, Kyushu University in Fukuoka, Japan. These were dissected on ice, and the fat bodies, midguts, and silk glands were collected and kept at -80° C until used. Total RNAs were rapidly extracted from the tissues dissected with Sepasol-RNA 1 (Nacalai Tesque, www.nacalai.co.jp) according to the manufacturer's instructions.

### Cloning and sequencing of the cDNA encoding of MCM7

Total RNA isolated from the fat body of the larvae was subjected to reverse transcriptase-polymerase chain reaction (RT-PCR). First-strand cDNA was produced using SuperScript II reverse transcriptase (Invitrogen, www.invitrogen.com) with an oligo-dT primer. The resulting cDNA was used as a template to amplify a DNA fragment by PCR with the following oligonucleotide primers: 5′-AACACCATGGCAATGCGTGAT-3′ (sense) and 5′-CCCAAGCTTTCACATGAA CGT-3′ (antisense) for MCM7. The underlined and double-underlined regions are *Nco*I and *Hind*III restriction enzyme sites, respectively. The primers were designed based on the partial sequence obtained from the SilkBase EST database (Mita et al. 2004) for the purpose of subcloning the PCR product into an expression plasmid vector. The PCR was conducted with one cycle at 94° C for 2 min, 35 cycles at 94° C for 1 min, 54° C for 1min, 72° C for 2 min, followed by one cycle at 72° C for 10 min. The PCR products obtained were ligated into pGEM-T Easy vector (Promega, www.promega.com). DNASIS software (version 3.4) was used for the sequence analysis. Homology alignment was performed by CLUSTALW (version 1.83), with 10 and 0.2 as the values of the gap creation penalty and gap extension, respectively. Preparation of the phylogenetic tree was done by Neighbour-joining plot software (http://www-igbmc.ustrasbg.fr/Bioinfo/ClastulX/Top.html).

### Overexpression and purification of recombinant proteins

The amplified products were cloned into pGEM-T Easy vector, as described above. After digestion of the PCR product with appropriate restriction enzymes, the fragment obtained was subcloned into the expression vector pET-28a for MCM7 (Novagen, www.emdchemicals.com). These prepared expression plasmids were transformed into competent *Escherichia coli* Rosetta (DE3) pLysS cells (Novagen), which were grown at 37° C on Luria-Bertani media containing 100 µg/ml ampicillin. After the cell density reached 0.7 OD_600_, isopropyl 1-thio-β-D-galactoside (IPTG) was added to the final concentration of 1 m*M* to induce the production of recombinant proteins. After further incubation for 3 h, cells were harvested by centrifugation, homogenized in a 20 m*M* Tris-HCl buffer (pH 8.0) containing 0.5 *M* NaCl, 4 mg/ml of lysozyme and 1 m*M* phenylmethanesulfonyl fluoride and disrupted by sonication. The supernatant was clarified by centrifugation at 10,000 × g for 15 min. An SDS-PAGE was conducted with a 15% Polyacrylamide slab gel containing 0.1% SDS according to the method of Laemmli (1970). Protein samples (10 µl) were mixed with the same volume of a 0.2 *M* Tris-HCl buffer (pH 6.8) containing 2% SDS, 2% 2-mercaptoethanol, 20% glycerol, and 2 × 10 ^-3^% bromophenol blue and boiled for 3 min. Protein bands were visualized by staining with Coomassie Brilliant Blue R250.

## Results and Discussion

### Cloning and sequencing of cDNA encoding of *B. mori* MCM

**Figure 1.**
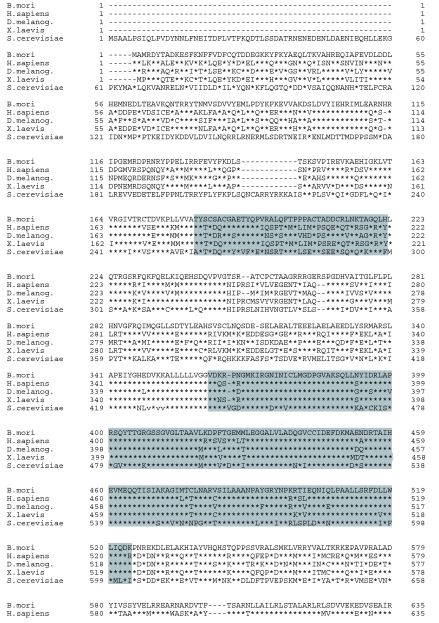
Alignment of amino acid sequences of MCM7. Sequences of MCM7s from different organisms were from Swiss-Prot and NCBI databases: *Bombyx mori* (determined in the present study); *Saccharomyces cerevisiae* (no. P38132); *Homo sapiens* (no. P33993); *Drosophila melanogaster* (no. AB010109); *Xenopus laevis* (no. Q91876). Zn-finger motif (184–222) and DNA-dependent ATPase morif (362–524) are shaded. High quality figures are available online.

**Figure 2.**
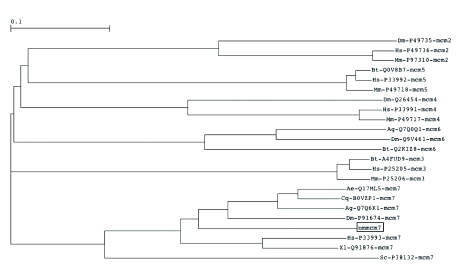
Phylogenetic analysis of amino acid sequences of MCM7. The phylogenetic tree was created with Neighbour-joining plot software. The citation number of each MCM was obtained from DDBJ and Swiss-Prot database. Numbers attached indicate branch length. Dm, *Drosophila melanogaster,* Hs, *Homo sapiens; Mm, Mus musculus*; Bt, *Bos taurus*; Ag, *Anopheles gambiae*; Ae, Aedes *aegypti*; Cq, *Culex quinquefasciatus;* bm, *Bombyx mori*; Xl, Xenopus laevis; Sp, *Saccharomyces cerevisiae.* High quality figures are available online.

The cDNA encoding the putative MCM was obtained by RT-PCR using total RNA from *B. mori* (p50 strain). The nucleotide sequence of the MCM was determined and deposited in GenBank under Accession No. AB177622. It contained an open reading frame of 2,160 bp, encoding 719 amino acid residues (Figure 1), whose theoretical molecular mass and pI were found to be 81,109 and 6.64, respectively. The deduced amino acid sequence of this putative MCM showed 69.5, 65.8, 64.3 and 44.2% identities to MCM7s from *Drosophila melanogaster, Xenopus laevis, Homo sapiens,* and *Saccharomyces cerevisiae,* respectively. Based on the phylogenetic tree generated from the aligned amino acid sequences of other MCM family proteins, the present MCM was included in the group of MCM7 and was closest to MCM7 of *D. melanogaster* (Figure 2). The complete genome sequences of several prokaryotes showed that no MCM-like sequences are found in eubacteria. MCM members belong to the AAA+ ATPase family. All known MCMs contain this motif, the MCM-box (VVCIDEFDKMSDMDRTA), which is embedded in a highly conserved domain in the middle of the MCM proteins (Hu et al. 1993; Musahl et al. 1995). The MCM-box contains the Walker A ATPase motif and the Walker B ATPase motif (Ishimi 1997). From this, the conclusion was that it was a member of the MCM7 family, bmMCM7. The central DEFDKM-peptide sequence is highly conserved among all known MCMs. The homologue of the MCM-box was found in the sequence of bmMCM7 between Va141 and Ala57 (Figure 1). bmMCM7, like other MCM proteins, possessed a putative Zn-finger motif (Thr184-His222) and a DNA-dependent ATPase (Val362-Lys524) motif, which are highly conserved in the central region of the amino acid sequence of MCM7. The ATPase plays an important role in initiation of DNA replication.

**continued:**
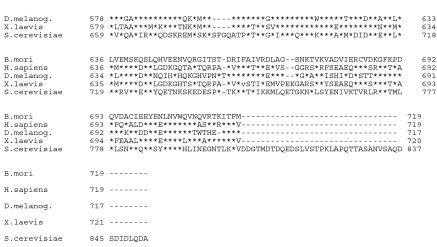

### Localization of the bmMCM7 transcript

**Figure 3.**
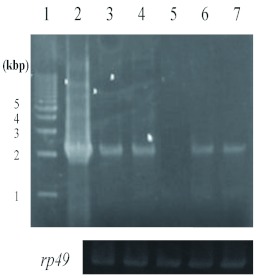
Localization of the MCM 7 transcript. First-strand cDNAs were synthesized with mRNAs from various tissues. RT-PCR was performed with the primers described in Materials and Methods. Lane 1, size marker; lane 2, positive control (pGEM-T Easy vector containing bmMCM7 cDNA); lane 3, ovaries; lane 4, testes; lane 5, midguts; lane 6, silk glands; lane 7, fat bodies. *rp49* was used as a loading control. High quality figures are available online.

Knowledge of the tissue distributions of bmMCM7 mRNA could help understand its physiology. The ribosomal protein 49 gene (*rp49*) was used as an endogenous control to normalize the expression of target genes. As shown in Figure 3, the RT-PCR revealed only a single band of approximately 2.0-kilo bases detected with the same migration with the band in positive control, which was obtained in RNA samples from the ovaries, testes, silk glands, and fat bodies. Small amounts of the mRNA were detected in the midgut. MCMs may be abundant in the proliferating tissues. The fifth-instar larvae of the silkworm grows rapidly, and each internal tissue is proliferated and differentiated at this stage. This result is consistent with a previous report (Ohno et al. 1998), showing that mRNAs encoding *D. melanogaster* MCMs were expressed at high levels during embryogenesis. The question why there was no bmMCM7 mRNA in the midgut remains unanswered. Further understanding of physiology of these proteins requires comprehensive studies on the developmental changes in activity, proteins, and mRNAs in various tissues.

### Attempt to overproduce the recombinant proteins

**Figure 4.**
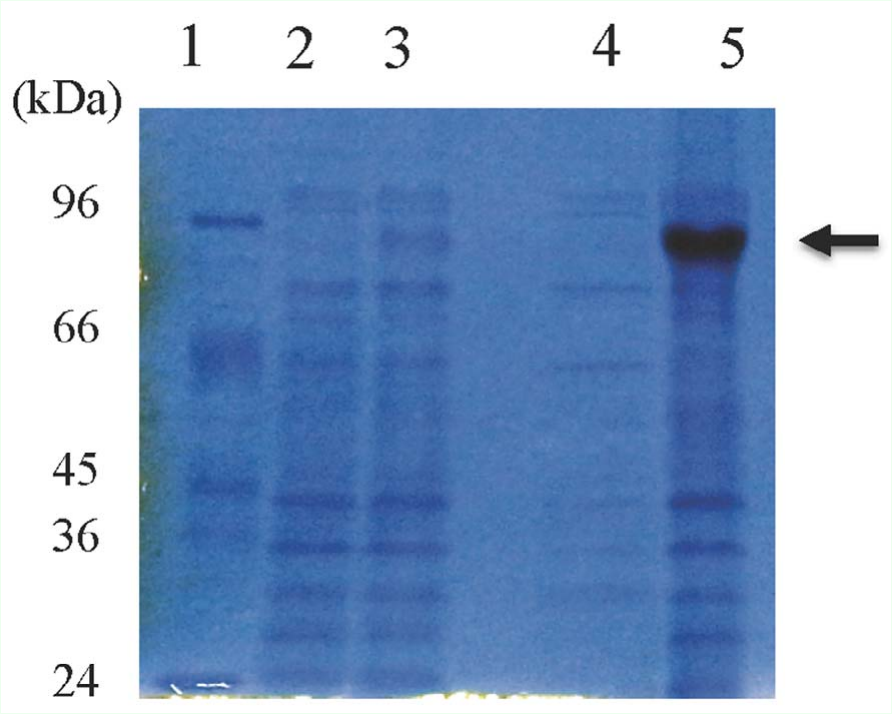
Electropherogams of rbmMCM7 overexpressed in *Escherichia coli* cells. *E. coli* cell extracts or purified protein were subjected to 15% SDS-PAGE followed by staining with Coomassie Brilliant Blue R250. Lane 1, protein molecular size markers; lane 2, extract of *E. coli* cells carrying the bmMCM7 expression vector without IPTG induction; lane 3, the same with IPTG induction; Lane 4, supernatant fraction; lane 5, inclusion body. High quality figures are available online.

The MCM family plays roles in many biological processes such as transcriptional activation, chromosome condensation, and recombination (Forsburg 2004). Especially, the replicative DNA helicase is formed by six MCMs (Forsburg 2004). All MCMs are members of the AAA+ protein family. We showed there was consensus among the sequences for ATP binding and hydrolysis in bmMCM7. To gain a better understanding of the role and structure of bmMCM7, it is necessary to obtain a large amount of the protein. The recombinant bmMCM7 (rbmMCM7) was overexpressed with a bacteriophage T7*lac* promoter in the *E. coli* expression vector, which was under the control of the *lac* repressor and induced by IPTG. After induction with IPTG, the cultured cells were collected and lysed with lysozyme and sonication. SDS-PAGE analysis of the *E. coli* cell lysate revealed that the extract gave an overproduced band, and it was in an insoluble form (Figure 4). The expressed protein migrated with an apparent molecular weight of 80,000 (Figure 4) was in agreement with the molecular weight from amino acid sequences. When the extract after lysis was centrifuged, the recombinant protein was present in the *E. coli* inclusion bodies. The inclusion bodies containing the recombinant proteins were solubilized with 8 *M* urea, and the solution was dialyzed to decrease the denaturant concentration to 4 *M*, 2 *M*, and finally 0 *M.* However, it was not possible to obtain the soluble form of the recombinant protein (data not shown). It is possible that the denatured proteins were refolded incompletely during the dialysis. More sophisticated procedures for the preparation of soluble rbmMCM7, instead of those involving inclusion bodies are to be examined. To improve the production of the protein, we are constructing a more efficient expression system of bmMCM7. Studies along these lines are in progress in our laboratories. The preparation of recombinant bmMCM7 in large amounts will contribute to detailed structural and biological studies of bmMCM7.

## Abbreviations

ARS: autonomous replication sequences;
IPTG: isopropyl 1-thio-β-D-galactoside;
MCM: a minichromosome maintenance protein;
RT-PCR: reverse transcriptase-polymerase chain reaction
